# Silibinin Regulates Lipid Metabolism and Differentiation in Functional Human Adipocytes

**DOI:** 10.3389/fphar.2015.00309

**Published:** 2016-01-21

**Authors:** Ignazio Barbagallo, Luca Vanella, Maria T. Cambria, Daniele Tibullo, Justyna Godos, Laura Guarnaccia, Agata Zappalà, Fabio Galvano, Giovanni Li Volti

**Affiliations:** ^1^Biochemistry Section, Department of Drug Science, University of CataniaCatania, Italy; ^2^Department of Biomedical and Biotechnological Sciences, University of CataniaCatania, Italy; ^3^Department of Surgery, University of CataniaCatania, Italy

**Keywords:** silibinin, brown adipocyte, thermogenesis, adipocyte, lipid metabolism, stem cells differentiation, human adipose tissue derived mesenchymal stem cells

## Abstract

Silibinin, a natural plant flavonolignan is the main active constituent found in milk thistle (*Silybum marianum*). It is known to have hepatoprotective, anti-neoplastic effect, and suppresses lipid accumulation in adipocytes. Objective of this study was to investigate the effect of silibinin on adipogenic differentiation and thermogenic capacity of human adipose tissue derived mesenchymal stem cells. Silibinin (10 μM) treatment, either at the beginning or at the end of adipogenic differentiation, resulted in an increase of SIRT-1, PPARα, Pgc-1α, and UCPs gene expression. Moreover, silibinin administration resulted in a decrease of PPARγ, FABP4, FAS, and MEST/PEG1 gene expression during the differentiation, confirming that this compound is able to reduce fatty acid accumulation and adipocyte size. Our data showed that silibinin regulated adipocyte lipid metabolism, inducing thermogenesis and promoting a brown remodeling in adipocyte. Taken together, our findings suggest that silibinin increases UCPs expression by stimulation of SIRT1, PPARα, and Pgc-1α, improved metabolic parameters, decreased lipid mass leading to the formation of functional adipocytes.

## Introduction

Obesity is characterized by hypertrophic adipocytes producing low adiponectin and increased tumor necrosis factor α (TNF-α) levels, which are associated with insulin resistance (Cantley, 2014; Moreno-Indias and Tinahones, 2015). On the other hand, adipose tissue can also suppress weight gain and metabolic disease through the action of specialized, heat-producing adipocytes (Harms and Seale, 2013). To date, two types of adipose tissue are known to exist: white (WAT) and brown (BAT) adipose tissue. WAT is widely distributed throughout the body, whereas BAT is present only in limited parts of the body such as interscapular, axillary, superior cervical, and perirenal regions. The physiological significance of these adipose tissues is completely different: WAT functions to store energy excess as fat, whereas BAT serves to consume energy excess as heat (thermogenesis) (Kajimura and Saito, 2014). Furthermore, brown adipocytes contain more capillaries compared to white adipocytes and their brown color is due to the presence of many mitochondria in the cell (Nam and Cooper, 2015).

In brown fat, the heat-generating pathway is the futile cycle of proton pumping through the actions of uncoupling protein 1 (UCP1) (Rousset et al., 2004). In cells expressing UCP1, the oxidation of lipids and carbohydrates results in the extraction of high-energy electrons, which flow down the electron transport chain (ETC) as protons are pumped across the inner mitochondrial membrane. Thus, much of the chemical energy generated by substrates oxidation in brown fat cells triggers a futile proton cycle leading to heat generation (Shabalina et al., 2013).

The biomedical interest in brown adipocytes focused on the capacity of these cell types to counteract metabolic disease, including obesity and type 2 diabetes (Poher et al., 2015). Brown adipocytes are located in specific areas and express constitutively high levels of thermogenic genes, whereas inducible ‘brown-like’ adipocytes, also known as beige cells, have a white fat phenotype and produce heat in response to various activators. Many genes and pathways regulating brown and beige adipocyte biology have now been identified, providing a variety of promising therapeutic targets for metabolic disease (Masella et al., 2012; Cohen and Spiegelman, 2015). Several natural and synthetic compounds have been shown to be actively involved in such pathways and among these, silibinin seems to be a very promising agent. Recent studies have demonstrated that silibinin, a natural plant flavonolignan has an anti-adipogenic effect on 3T3-L1 cells (Kim et al., 2009) and suppresses lipid accumulation in adipocytes. In particular, a previous report showed that silibinin regulates underlying signaling for hypertrophy and hyperplasia in adipocyte, and anti-adipogenic effect of silibinin was exerted on early adipogenic stage during adipogenesis via cell cycle arrest (Suh et al., 2015). Most of the studies utilized murine 3T3-L1 cell line for examination of adipocyte development as these cells readily accumulate lipid upon differentiation (Christy et al., 1989; Ntambi and Young-Cheul, 2000; Burton et al., 2002). However, with the discovery of human adipose tissue derived mesenchymal stem cells (ASCs; Zuk et al., 2001), this multi-potent lineage has become the main focus for analyzing changes during differentiation (DeLany et al., 2005; Yu et al., 2010).

Objective of this study was to investigate the effect of silibinin on adipogenic differentiation and thermogenic capacity of human adipose tissue derived mesenchymal stem cells.

## Materials and Methods

### Adipose Stem Cells Isolation and Culture

Adipose tissue sample was obtained from a patient underwent abdominal plastic surgery (male, 30 years old, 98 kg of body weight); the subject provided his written consent before inclusion in the study. Since this is a non-therapeutic trial, it was carried out with the consent of the subject legally acceptable according our Italian Government (Legge 675/1996 and DL 196/2003, art. 40. Art 32 Codice Italiano di Deontologia Medica). Adipose tissue was minced with scissors and scalpels into less than 3-mm pieces and isolation of ASCs proceeded as previously described (Salomone et al., 2013). Briefly, after gentle shaking with equal volume of PBS, the mixture separated into two phases. The upper phase (containing stem cells, adipocytes, and blood) after washing with PBS was enzymatically dissociated with 0.075% collagenase (type I)/PBS for 1 h at 37°C with gentle shaking. The dissociated tissue was then mixed with an equal volume of DMEM (GIBCO-BRL, Japan) supplemented with 10% FBS and incubated 10 min at room temperature. The solution then was separated into two phases. The lower phase was centrifuged at 1500 rpm for 5 min at 20°C. The cellular pellet was resuspended in 160 mM NH_4_Cl to eliminate erythrocytes and passed through a 40 μm mesh filter into a new tube. The cells were resuspended in an equal volume of DMEM/10% FBS and then centrifuged. Isolation resulted in obtaining 7.7 × 10^6^ of adherent cells for a primary culture from 5 g of adipose tissue (approximately; 1.0 × 10^5^ to 4.6 × 10^6^/1 g) after 7 to 10 days of culture. The cells were suspended in DMEM/10% FBS plated in concentration 1–5 × 10^6^ cells/75 cm^2^. The phenotype of ASCs was evaluated by flow-cytometry analysis (FC500 Beckman Coulter). The ASCs presented as a homogeneous fibroblastic cell population. Flow-cytometric analysis of passage 4th cells revealed that cells were negative for CD34 and CD45, and that cells were positive for CD105 and CD90 (Data not shown).

### Differentiation of Human ASCs into Adipocytes

Adipose tissue derived mesenchymal stem cells (passage 4 to 5) were plated in a 75-cm^2^ flask at a density of 1 to 2 × 10^4^ cells and cultured in DMEM with 10% FBS for 7 days. The medium was replaced with adipogenic medium, and the cells were cultured for an additional 7 or 14 days.

The adipogenic media consisted of complete culture medium supplemented with DMEM-F12 high glucose, 3% (v/v) FBS, 100 nM insulin, 100 nM dexamethasone (Sigma–Aldrich, St. Louis, MO, USA), 0.5 mM isobutylmethylxanthine (Sigma–Aldrich, St. Louis, MO, USA), 60 μM indomethacin (Sigma–Aldrich, St. Louis, MO, USA) and transferrin 10 μg/ml. Media were changed every 3 days. Human ASCs were cultured in the presence of Silibinin (10 μM) which was administered every 3 days in the first set of experiments while for the second set of experiments Silibinin was added for 24 h after 14 days of adipogenic differentiation.

### Oil Red O staining

Staining was performed using 0.21% Oil Red O in 100% isopropanol (Sigma–Aldrich, St. Louis, MO, USA). Briefly, adipocytes were fixed in 10% formaldehyde, stained with Oil Red O for 10 min, rinsed with 60% isopropanol (Sigma–Aldrich), and the Oil Red O eluted by adding 100% isopropanol for 10 min and the optical density (OD) measured at 490 nm, for 0.5 s reading. Lipid droplets accumulation was examined by using inverted multichannel LED fluorescence microscope (Evos, Life Technologies, Grand Island, NY, USA).

### RNA Extraction and qRT-PCR

RNA was extracted by Trizol reagent (Invitrogen, Carlsbad, CA, USA). First strand cDNA was then synthesized with Applied Biosystem (Foster City, CA, USA) reverse transcription reagent.

Quantitative real-time PCR was performed in 7900HT Fast Real-Time PCR System Applied Biosystems using the SYBR Green PCR MasterMix (Life Technologies, Milan, Italy). The primer sequences used are shown in **Table 1**. The specific PCR products were detected by the fluorescence of SYBR Green, the double stranded DNA binding dye. The relative mRNA expression level was calculated by the threshold cycle (Ct) value of each PCR product and normalized with that of GAPDH by using comparative 2^-ΔΔCt^ method.

**Table 1:** PCR primers used in this study.

Gene	Primer forward	Primer reverse
Adiponectin	AGGCTTTCCGGGAATCCAAG	CGCTCTCCTTCCCCATACAC
DGAT1	CGCGGACTACAAATGGACGA	AACCAGTAAGACCACAGCCG
DLK1	TCCTCAACAAGTGCGAGACC	CTGTGGGAACGCTGCTTAGA
FABP4	AAACTGGTGGTGGAATGCGT	GCGAACTTCAGTCCAGGTCA
FAS	CGGAGGCATCAACCCAGATT	GATGGTGGTGTAGACCTTCCG
GAPDH	AGACACCATGGGGAAGGTGA	TGGAATTTGCCATGGGTGGA
IL6	CTTCTCCACAAGCGCCTTCG	CTGGCATTTGTGGTTGGGTC
IRS1	GCAACCAGAGTGCCAAAGTG	AGGTCATTTAGGTCTTCATTCTGCT
PGC1α	GGTGCAGTTTTGCCAAGGAG	TTCCTTGGGGTCCAGACAGA
PPARα	AAGAGCTTGGAGCTCGGC	TGAAAGCGTGTCCGTGATGA
PPARγ	AGAGTACGTGGGAGAAATGAC	GATGGCCACCTCTTTGCTCT
SIRT1	TGATTGGCACAGATCCTCGAA	AAGTCTACAGCAAGGCGAGC
TNF α	CTCGAGTCAGATCATCTTCTCGCACCCCG	GGAATTCTGTTCGTCCTCCTCACAGGGC
UCP-1	TGTCCTGGGAACAATCACCG	TCCAGGATCCAAGTCGCAAG
UCP-2	GCCTCTACAATGGGCTGGTT	GAGCATGGTAAGGGCACAGT
UCP-3	AGCCCCCTCGACTGTATGAT	ACTTTCATCAGGGCCCGTTT

### Western Blot Analysis

Western Blot analysis was performed as previously described (Vanella et al., 2013). Primary polyclonal antibodies directed against UCP-1 and beta-actin were purchased from Santa Cruz Technologies. Protein detection was carried out using a secondary infrared fluorescent dye conjugated antibody absorbing at 800 nm or 700 nm. The blots were visualized using an Odyssey Infrared Imaging Scanner (Li-Cor Science Tec) and quantified by densitometric analysis performed after normalization with b-actin.

### Statistical Analysis

Statistical significance (*P* < 0.05) of differences between experimental groups was determined by the Fisher method for analysis of multiple comparisons. For comparison between treatment groups, the null hypothesis was tested by either single-factor analysis of variance (ANOVA) for multiple groups, or the unpaired *t*-test for two groups, and the data are presented as mean ± SEM.

## Results

### Analysis of Adipogenic Differentiation

**FIGURE 1:**
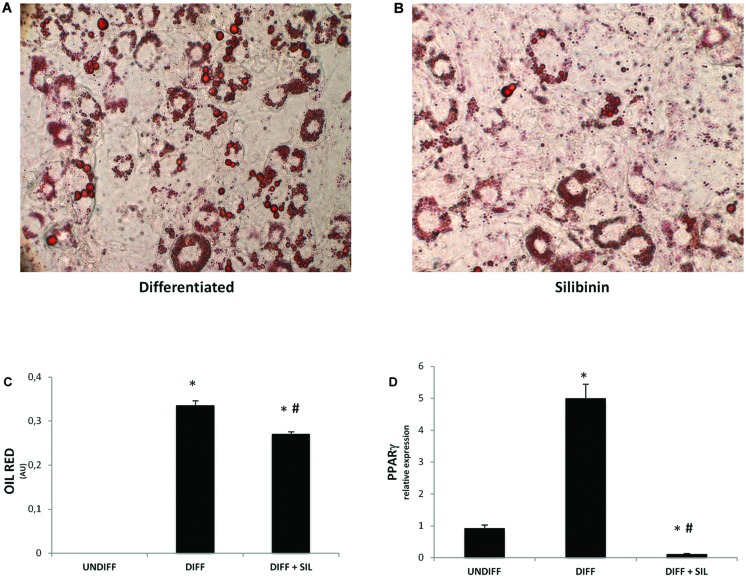
**(A–B)** Lipid droplets accumulation measured by Oil red staining in differentiated cells in presence **(B)** or absence of silibinin **(A)**. **(C)** Oil Red staining measured by spectrophotometer (λ = 490 nm) in undifferentiated cells and after 14 Days of adipogenic differentiation in presence or absence of silibinin. **(D)** PPARγ gene expression was evaluated by Real Time PCR. All values are expressed as mean ± SEM of four experiments (*n* = 4) in duplicate. ^∗^*P* < 0.05 vs. undifferentiated; #*P* < 0.05 vs. differentiated.

**FIGURE 2:**
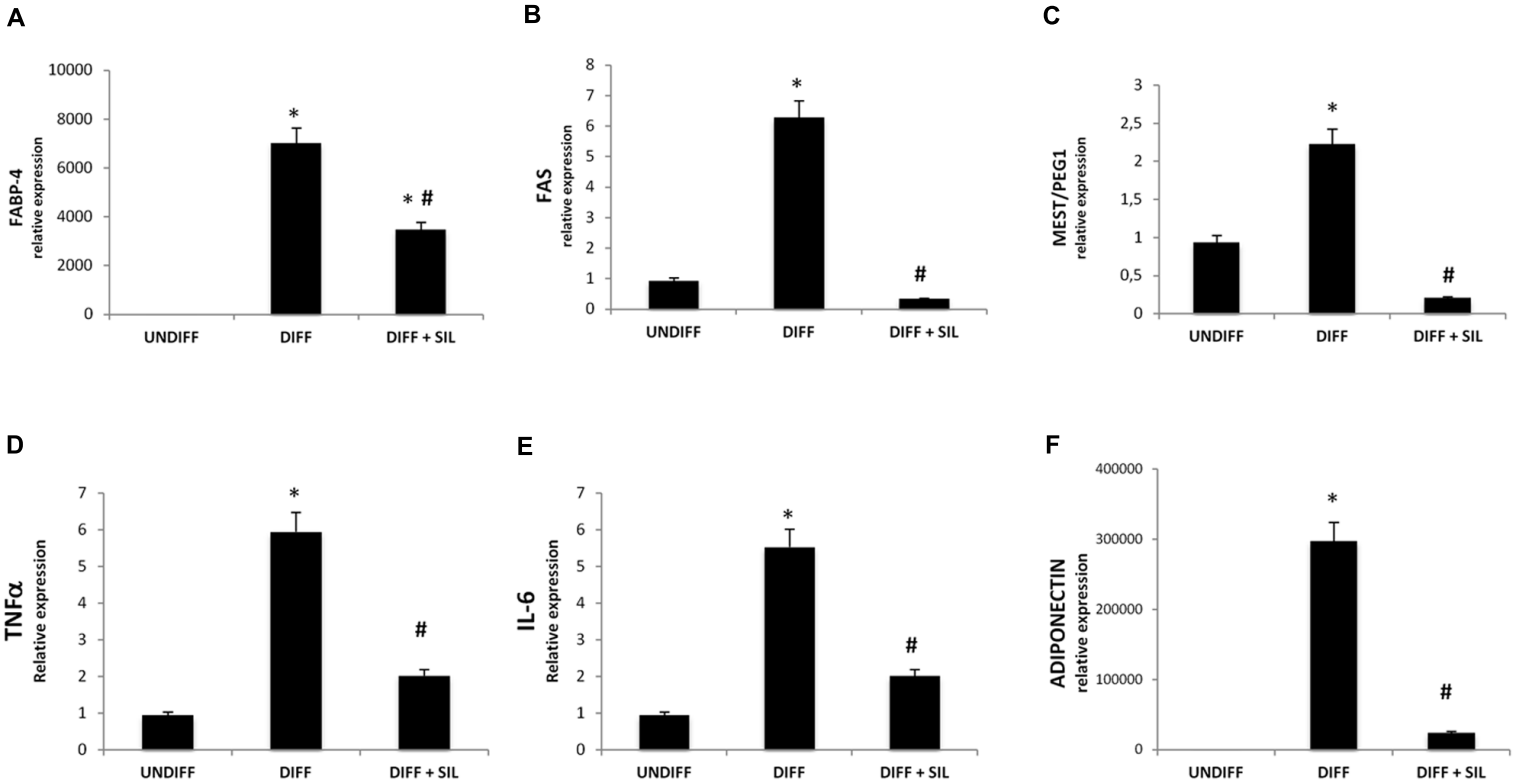
**Analysis of gene expression by Real time PCR of FABP4 (A), FAS (B), MEST/PEG1 (C), TNFα (D), IL-6 (E), and adiponectin (F).** All values are expressed as mean ± SEM of four experiments (*n* = 4) in duplicate. ^∗^*P* < 0.05 vs. undifferentiated; #*P* < 0.05 vs. differentiated.

To investigate signals that might regulate the differentiation of ASCs, we analyzed the mRNA levels of peroxisome proliferator-activated receptor gamma (PPARγ; **Figure 1D**), fatty acid binding protein 4 (FABP4; **Figure 2A**), fatty acid synthase (FAS; **Figure 2B**) and mesoderm-specific transcript (MEST/PEG1; **Figure 2C**). We showed that all of these markers resulted in a significantly increase after 14 days of adipogenic differentiation. In addition, **Figures 1A–C** shows positive Oil Red staining of the cells following 14 days of differentiation.

### The Effect of Silibinin on the Adipogenesis

Quantification of Oil Red stained cells showed that lipid droplets decreased following Silibinin treatment (**Figures 1A,B**). As seen in **Figure 1C** PPARγ was significantly reduced by silibinin treatment respect to the differentiated cells. Moreover, the administration of silibinin during the adipogenic differentiation was able to reduce significantly the mRNA levels of PPARγ (**Figure 1C**), FABP4 (**Figure 2A**), FAS (**Figure 2B**), MEST/PEG1 (**Figure 2C**), and Adiponectin (**Figure 2F**).

### Silibinin Reduces TNF-α and Il-6 Expression

In order to study the potential anti-inflammatory effects of silibinin, we investigated TNF-α and IL-6 expression during differentiation. We show a significantly increase of mRNA levels of these cytokines in differentiated adipocytes (**Figures 2D,E**). Silibinin treatment was able to decrease significantly TNF-α and IL-6 mRNA levels in differentiated cells.

### Effect of Silibinin on Thermogenic Genes Expression

**FIGURE 3:**
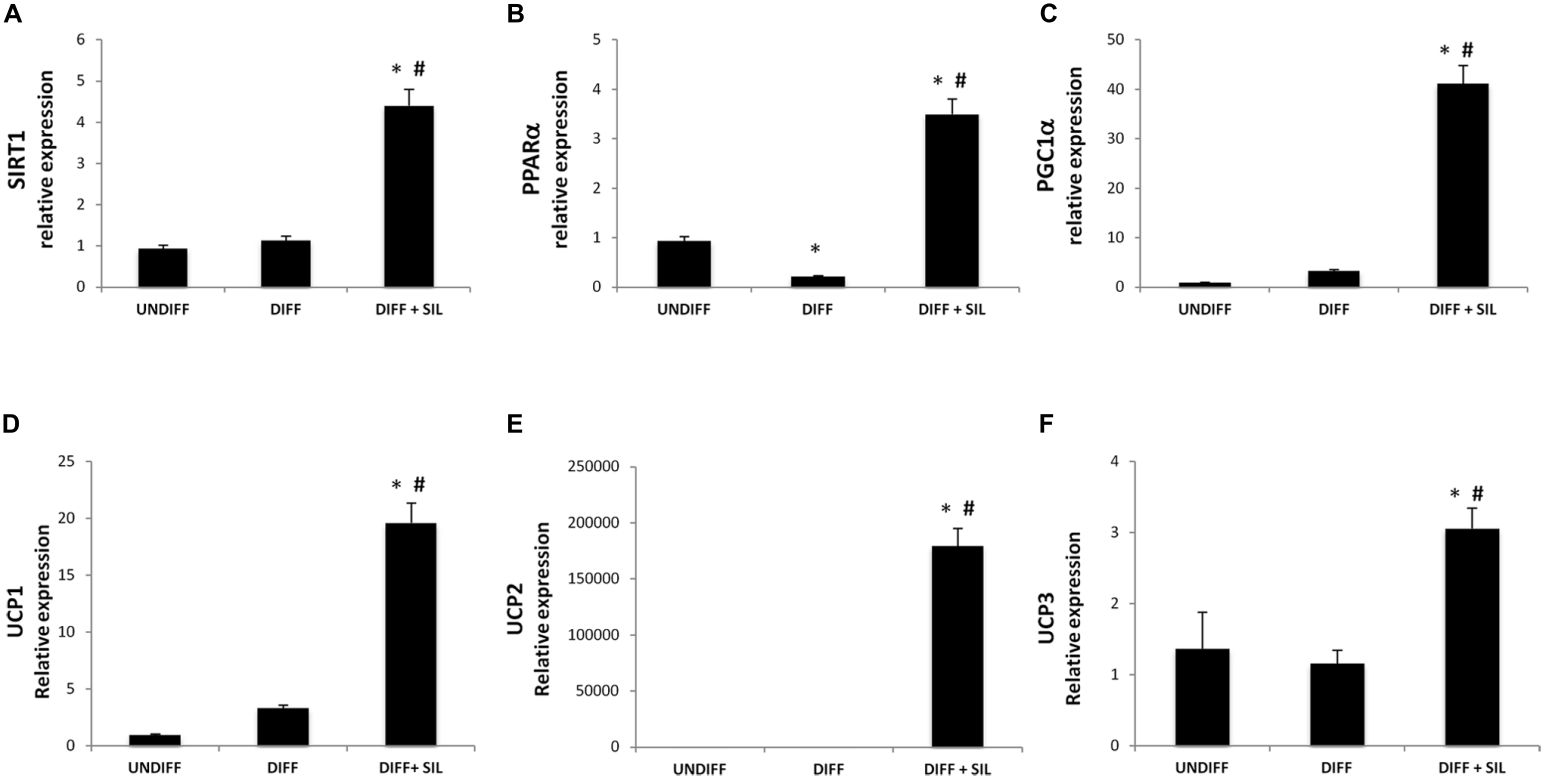
**Analysis of gene expression by Real time PCR of SIRT1 (A), PPARα (B), PGC1α (C), UCP1 (D), UCP2 (E), and UCP3 (F).** All values are expressed as mean ± SEM of four experiments (*n* = 4) in duplicate. ^∗^*P* < 0.05 vs. undifferentiated; #*P* < 0.05 vs. differentiated.

In order to investigate the effect of silibinin on lipid metabolism, we analyzed the expression of the thermogenic pathway markers. The administration of Silibinin during adipogenic differentiation was able to significantly increase mRNA levels of sirtuin 1 (SIRT-1), peroxisome proliferator-activated receptor alpha (PPARα) and peroxisome proliferator-activated receptor gamma, co-activator 1 alpha (Pgc-1α) (**Figures 3A–C**). Moreover, to study the activation of heat-generating pathway, which is the futile cycle of proton pumping through the actions of UCPs, we analyzed the expression of UCP1, UCP2, and UCP3. These set of experiments showed that Silibinin was able to significantly increase the expression levels of UCP1, 2, and 3 (**Figures 3D–F**).

### The Effect of Silibinin on Mature Adipocytes

**FIGURE 4:**
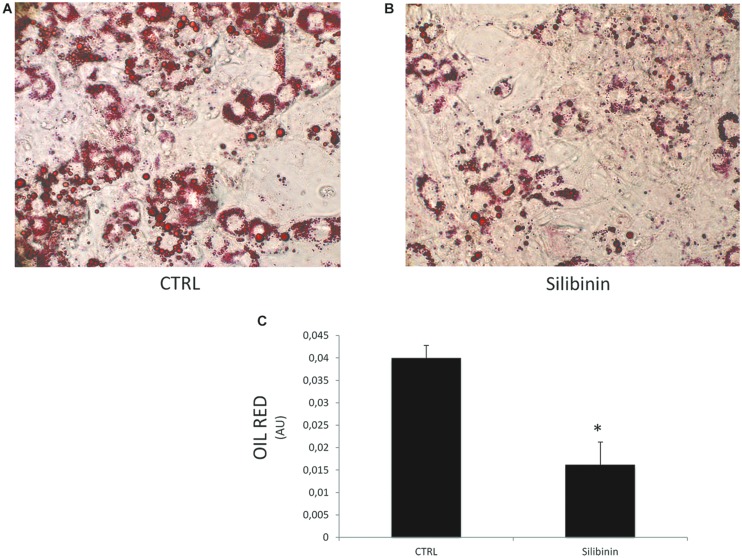
**Lipid droplets accumulation measured by Oil red staining in differentiated cells CTRL (A) and in differentiated cells treated for 24 h with silibinin (B).** Oil Red staining measured by spectrophotometer (λ = 490 nm) in differentiated cells and after silibinin treatment **(C)**. All values are expressed as mean ± SEM of four experiments (*n* = 4) in duplicate. ^∗^*P* < 0.05 vs. CTRL.

**FIGURE 5:**
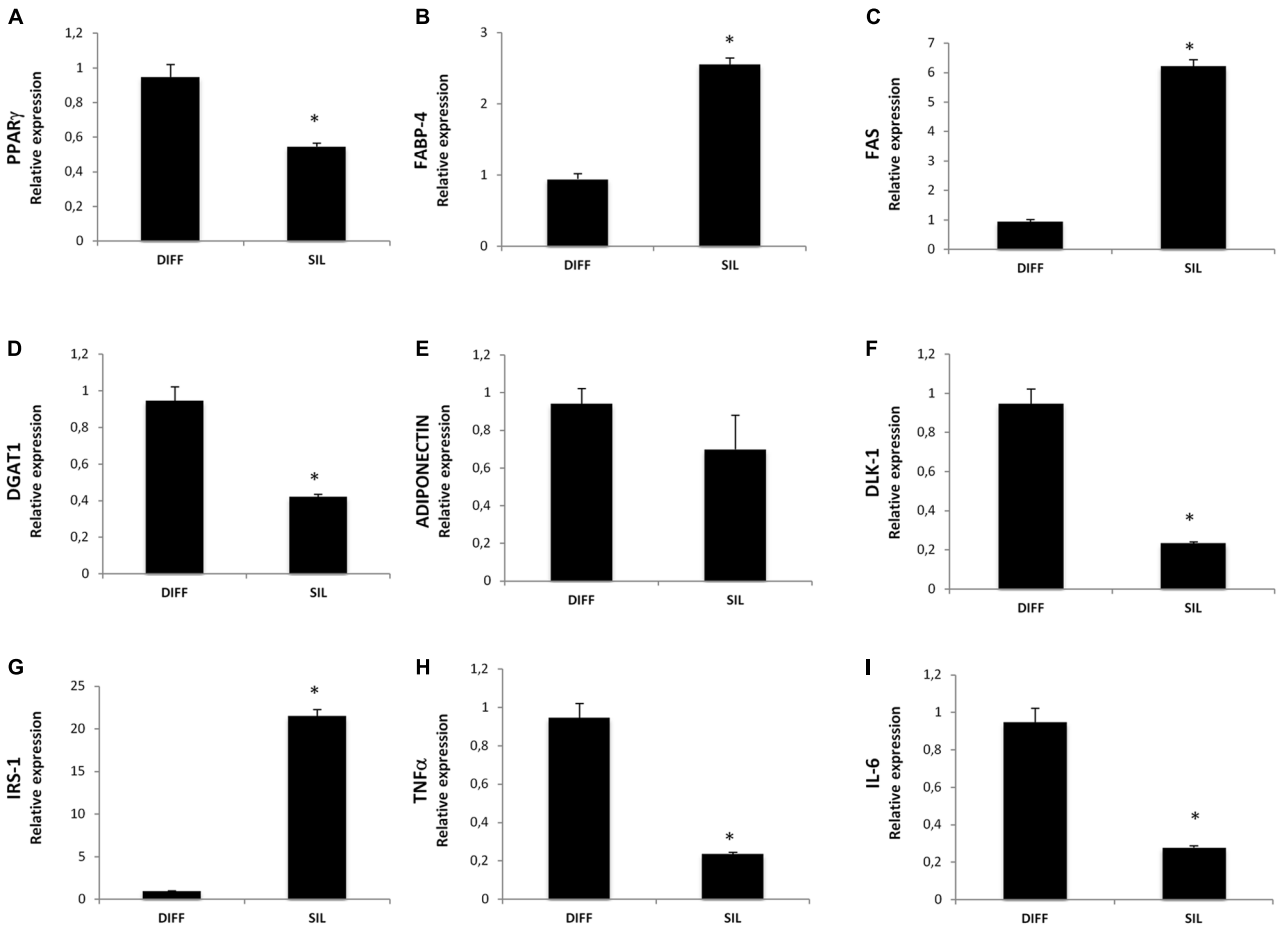
**Analysis of gene expression, evaluated by Real Time PCR, of cells treated with silibinin for 24 h after adipogenic differentiation.** Each graph **(A–I)** represents the relative expression of a single gene indicated on the Y axis compared to differentiated untreated cells. All values are expressed as mean ± SEM of four experiments (*n* = 4) in duplicate. ^∗^*P* < 0.05 vs. DIFF.

In a new set of experiments, we examined the effect of Silibinin on lipid accumulation after 14 days of differentiation by measuring Red Oil-stained lipid droplet area (**Figure 4**). Quantification of Oil Red stained cells showed that lipid droplets decreased following Silibinin treatment (24 h). Furthermore, we observed a significantly reduction of PPARγ (**Figure 5A**), diacylglycerol *O*-acyltransferase 1 (DGAT1; **Figure 5D**) and Delta like 1 (DLK-1) (**Figure 5F**) mRNA levels following silibinin treatment (**Figure 5A**). Interesting, silibinin showed a significantly increase of FABP4, FAS, and insulin receptor substrate 1 (IRS-1) genes expression (**Figure 5**).

Consistently with the first set of experiments, TNF-α and Il-6 resulted in a significantly decrease of mRNA levels following silibinin treatment.

### Silibinin Switches the Lipid Metabolism of Adipocyte Toward the Thermogenic Pathway

**FIGURE 6:**
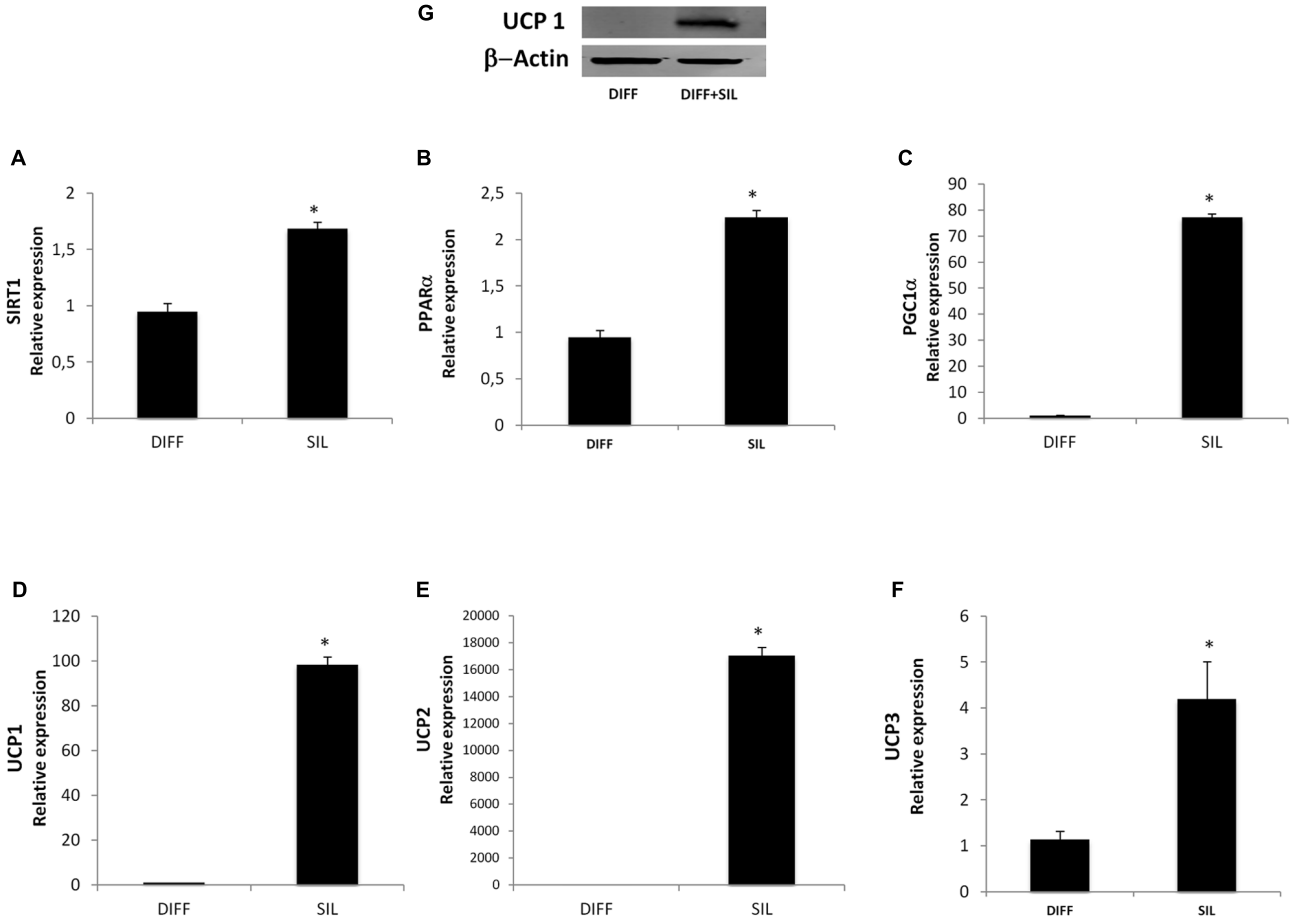
**(A–F)** Analysis of gene expression, evaluated by Real Time PCR, of cells treated with silibinin for 24 h after adipogenic differentiation. **(G)** Western Blot analysis of UCP-1 expression in differentiated adipocytes following silibinin treatment. All values are expressed as mean ± SEM of four experiments (*n* = 4) in duplicate. ^∗^*P* < 0.05 vs. DIFF.

We found that all markers of thermogenic pathway of brown adipocytes resulted in a significantly increase following silibinin treatment. In particular, in **Figure 6** we showed that silibinin administration for 24 h was able to increase the expression of SIRT-1, PPARα, Pgc-1α, UCP-1, UCP-2, and UCP-3. Moreover, the increased UCP-1 expression was further confirmed by Western Blot (**Figure 6G**).

## Discussion

Silibinin, a natural plant flavonolignan is the main active constituent found in milk thistle (*Silybum marianum*), a plant of the Asteraceae family. In particular, it is known to have hepatoprotective and anti-neoplastic effect (Li Volti et al., 2011; Marrazzo et al., 2011; Salamone et al., 2012). Ka et al. (2009) have shown that silibinin inhibits adipogenesis of 3T3 cells by promoting the expression of insulin-induced genes 1 and 2 (INSIG1 and INSIG2), which block activation of sterol regulatory element binding protein-1c (SREBP1-c) (Kim et al., 2009). Furthermore, Suh et al. (2015) identified anti-adipogenic effect of silibinin in zebrafish via the down-regulation of adipogenic factors and the reduction of lipid accumulation in adipocyte and it is thought to be due to the regulation of silibinin on the phosphorylation of AMP-activated protein kinase alpha (AMPKa) and Acetyl-CoA carboxylase (ACC) (Suh et al., 2015). Adipocyte differentiation and its impact on restriction or expansion of particular adipose tissue depots have physiological and pathophysiological significance in view of the different functions of these depots. Brown or “beige” fat expansion can enhance thermogenesis, lipid oxidation, insulin sensitivity, and glucose tolerance; conversely expanded visceral fat (VAT) is associated with insulin resistance, low-grade inflammation, dyslipidemia, and cardio metabolic risk.

In the present study we showed that silibinin treatment during and at the end of adipogenic differentiation of ASC cell induces thermogenesis pathway in adipocyte by activation of UCPs.

**FIGURE 7:**
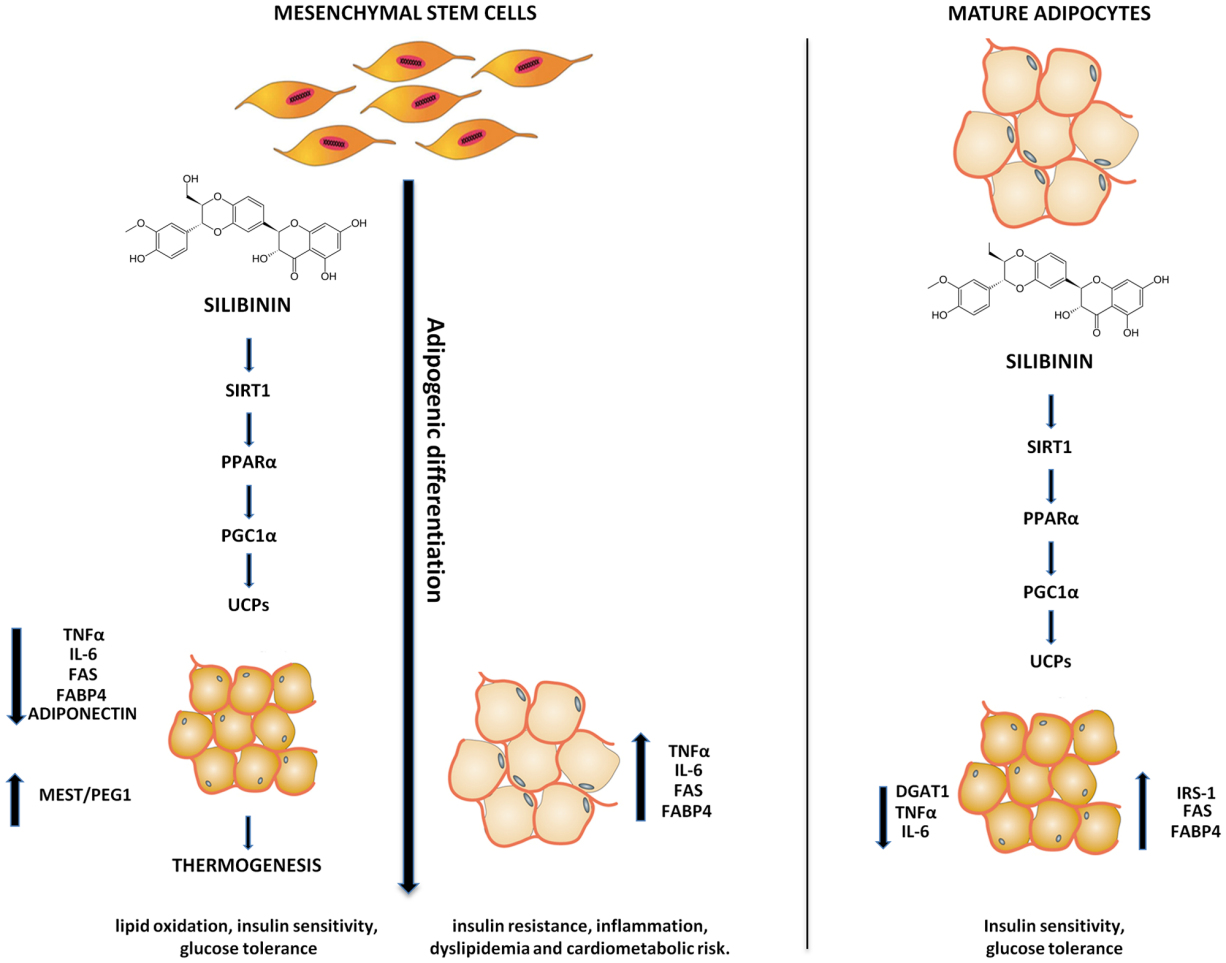
**Scheme of the silibinin effects during adipogenic differentiation and in mature adipocytes.** Adipogenic differentiation causes an increase in TNFα, IL-6, FAS, FABP4, and MEST/PEG1 resulting in an increase of insulin resistance, inflammation, dyslipidemia, and cardiometabolic risk. Silibinin switches the lipid metabolism of adipocyte to thermogenic pathway resulting in an improvement of insulin sensitivity and glucose tolerance.

Our data showed that silibinin is able to switch the lipid metabolism from white adipocytes phenotype to beige or brown. Silibinin treatment, from the beginning or at the end of adipogenic differentiation, resulted in an increase of SIRT-1, PPARα, Pgc-1α, and UCPs. On the other hand, the silibinin effects resulted in a decrease of PPAR gamma, FABP4, FAS, and MEST/PEG1 expression during the differentiation, confirming that its administration is able to reduce the fatty acid accumulation and the size of the cells. Moreover, decreasing in TNF-α and IL-6 expression in the differentiated adipocyte treated with silibinin respect the untreated adipocyte confirms a reduction of inflammation (**Figure 7**). Consistently with our findings previous reports showed that TNF-α inhibits SIRT1 expression in human adipocytes (Serrano-Marco et al., 2012). SIRT1, an NAD^+^ dependent type III deacetylase sirtuin, enhances glucose tolerance by potentiating brown adipose tissue function (Boutant et al., 2015) contributing to energy expenditure and browning of WAT and resistance to dietary obesity (Wang et al., 2013), interacts with PPARα and is required to activate PGC-1α (Purushotham et al., 2009). PPARα plays an important role in lipid metabolism, and activation of PPARα in human WAT leds to the appearance of brown fat gene expression, including UCP1 and PGC-1α (Mandard et al., 2004; Hondares et al., 2011). PPARα has been considered a distinctive marker of BAT with respect to the WAT phenotype (Villarroya et al., 2007). Finally, in this pathway, Pgc-1α is essential for cold-induced or β3-agonist-induced thermogenic activation of brown adipocytes (Uldry et al., 2006) and the expression of thermogenic genes in WAT (Kleiner et al., 2012). In addition, we found a significantly decrease of adiponectin expression in adipocytes differentiated with silibinin. These data are consistent with previously studies demonstrating that adiponectin overexpression significantly decreases UCP1 and PGC-1α protein levels (Qiao et al., 2014). In order to study the effects of silibinin on the mature adipocytes we also treated the cells at the end of differentiation. The mature adipocytes treated with silibinin (24 h) showed a significantly decrease of fatty acid accumulation and of DGAT1 gene expression, enzyme required for triacylglycerol synthesis and lipid droplets in adipocytes (Harris et al., 2011). In contrast to what was observed for silibinin treatment during the differentiation, FABP4 and FAS gene expression resulted in a significantly increase, which could be a response to lipolytic effect of silibinin. Moreover, the expression of those enzymes involved in lipid uptake and mobilization, favoring fatty acid utilization through uncoupled respiration (Teruel et al., 2005). Furthermore, we found an increase of IRS1 gene expression in adipocyte treated with silibinin. This data are consistent with a recent study showing that IRS-1 plays important roles in brown adipocyte differentiation where defects in differentiation in the IRS-1 knockout cells can be restored by reconstitution of these cells with IRS-1 (Tseng et al., 2004). In the present study, we also showed the decrease of DLK1 gene expression by silibinin in mature adipocytes. This gene is a molecular gatekeeper of adipogenesis which acts by maintaining the pre-adipocyte state and preventing adipocyte differentiation (Hudak and Sul, 2013). In addition, it was demonstrated that DLK-1 preferentially inhibits heat production in brown adipose tissue (Rakhshandehroo et al., 2012).

Our results showed that silibinin could induce thermoregulation improving metabolic homeostasis. In particular, silibinin modulated adipocytes lipid metabolism, inducing thermogenesis and promoting a brown remodeling of WAT. Taken together, our findings suggest that silibinin increases UCPs expression by stimulation of SIRT1, PPARα, and Pgc-1α, which might mediate the induction of higher energy efficiency, improved metabolic parameters, decreased fat mass and formation of functional adipocytes. In conclusion, silibinin may serves as a potential pharmacological tool to restore adipocyte function in metabolic diseases by acting as the biochemical switching of adipocytes phenotype.

## Author Contributions

IB and GL conceived and coordinated the study and wrote the paper. IB, LV, MC, DT, LG, AZ, and FG designed, performed, and analyzed the experiments. JG provided technical assistance and contributed to the preparation of the figures. All authors reviewed the results and approved the final version of the manuscript.

## Conflict of Interest Statement

The authors declare that the research was conducted in the absence of any commercial or financial relationships that could be construed as a potential conflict of interest.
